# Phototherapy for Skin of Color in the Biologic Era: A Cross-Sectional Survey of Moroccan Dermatologists’ Knowledge and Practice

**DOI:** 10.7759/cureus.112890

**Published:** 2026-07-17

**Authors:** Bouchra Baghad, Hayat Dahbi Skali, Soumiya Chiheb

**Affiliations:** 1 Department of Dermatology and Venereology, Ibn Rochd University Hospital, Hassan II University of Casablanca, Casablanca, MAR

**Keywords:** dermatology practice, morocco, phototherapy, skin of color, survey

## Abstract

Background

Phototherapy remains central to the treatment of inflammatory and pigmentary skin disease and is especially relevant in populations with darker skin phototypes. As biologic and targeted therapies expand, the place of phototherapy in resource-limited settings is uncertain. We assessed the knowledge, perceptions, and practice of Moroccan dermatologists regarding phototherapy and identified barriers to its use.

Methods

A cross-sectional, self-administered, web-based survey (34 items) was distributed to Moroccan dermatologists via the Moroccan Society of Dermatology network between October and November 2025, covering demographics, access, technical competency, knowledge, prescribing, attitudes toward biologic and targeted therapies, and training needs. Exploratory cross-tabulations (Fisher’s exact test) and 95% CIs (Clopper-Pearson method) were computed for the principal proportions.

Results

Of 53 respondents (91% female, 51% in private practice), 94% had ever prescribed phototherapy, but only 43% (95% CI: 30-58%) had access to a unit. Most respondents rated their knowledge of indications as sufficient or better; however, only 17% (95% CI: 8-30%) identified the guideline-based psoralen plus ultraviolet A risk-monitoring threshold, and 34% (95% CI: 22-48%) identified the recommended minimum age for narrowband ultraviolet B. Most (70%) considered phototherapy to retain a major role alongside biologic and targeted therapies, and a majority reported offering it before escalation in psoriasis (83%), vitiligo (76%), and atopic dermatitis (62%). Access to a phototherapy unit was strongly associated with public or university practice (p < 0.001) and with regular prescribing (p < 0.001). Logistical (79%), institutional (45%), and space-related (38%) barriers predominated; only 36% had received phototherapy training during residency.

Conclusions

Moroccan dermatologists express strong support for phototherapy and report offering it before biologic and targeted therapies across major indications, but deficits in equipment access, guideline-based knowledge, and postgraduate training represent barriers to optimal practice. These findings highlight the need for further research, including objective competency assessments and clinical outcome studies, to inform strategies for equipment deployment, structured training, and a national phototherapy network.

## Introduction

Phototherapy, encompassing narrowband ultraviolet B (NB-UVB), psoralen plus ultraviolet A (PUVA), and excimer light, has been a mainstay of dermatological treatment for more than five decades. Its efficacy across inflammatory, pigmentary, and lymphoproliferative disease is well established, and its low cost relative to systemic biologic and targeted therapies makes it especially valuable where resources are limited [1-3].

The Moroccan context makes phototherapy both particularly relevant and particularly challenging. Most Moroccans have Fitzpatrick phototypes III to V, that is, skin of color (SOC). Melanin-rich epidermis attenuates UV-induced erythema, raises minimal erythema dose thresholds, and alters the risk profile of UV therapy. Observational data suggest that the risk of PUVA-associated non-melanoma skin cancer, well established in phototypes I-II, may be lower in more deeply pigmented skin, although cumulative carcinogenic and photoaging risks remain, and long-term safety data specifically in phototypes III-V are limited [4,5]. Separately, vitiligo, for which NB-UVB is a cornerstone of management, carries a disproportionate psychosocial burden in SOC because of high lesional contrast between depigmented and normally pigmented skin [6]. It should be noted that ultraviolet exposure may exacerbate post-inflammatory hyperpigmentation in some patients, and these two conditions should not be regarded as equivalent phototherapy indications.

The rapid expansion of biologic and targeted therapies has raised concern that phototherapy may be displaced in routine practice. In psoriasis, IL-17 and IL-23 inhibitors have transformed the therapeutic landscape; in atopic dermatitis, dupilumab and JAK inhibitors (targeted small-molecule therapies, distinct from biologics) offer alternatives to phototherapy, and in vitiligo, topical ruxolitinib provides a targeted option [7,8]. This concern is particularly acute in low- and middle-income countries (LMICs), where the cost-effectiveness of phototherapy relative to these newer agents [3] may favor its use as a first-line approach.

Few data describe the actual state of phototherapy practice in Morocco: dermatologists’ technical knowledge, access barriers, and attitudes at the interface of phototherapy with biologic and targeted therapies. Using a cross-sectional survey, we sought to characterize the self-reported knowledge and practice of Moroccan dermatologists regarding phototherapy, identify the principal barriers to its use, and examine attitudes toward phototherapy in an expanding landscape of biologic and targeted therapies.

## Materials and methods

Study design and setting

We conducted a cross-sectional, observational survey of dermatologists practicing in Morocco between October and November 2025, coordinated by the Department of Dermatology, Ibn Rochd University Hospital (CHU Ibn Rochd), Casablanca. The study is reported in accordance with the STrengthening the Reporting of OBservational studies in Epidemiology (STROBE) statement for cross-sectional studies and with the Checklist for Reporting Results of Internet E-Surveys (CHERRIES) checklist for web-based surveys, where applicable.

Ethics statement

This study consisted of an anonymous and voluntary questionnaire-based survey conducted among Moroccan dermatologists. No patient data or identifiable personal information were collected. Participation was voluntary, and completion of the questionnaire implied informed consent. The study was conducted in accordance with the principles of the Declaration of Helsinki.

Survey instrument

A structured 34-item questionnaire was developed in French (the language of medical training in Morocco) and administered through Google Forms (Google LLC, Mountain View, CA, USA). Items were drawn from prior phototherapy surveys and piloted among five senior dermatologists for clarity and face validity. The instrument was not formally psychometrically validated; this is acknowledged as a limitation. Six domains were covered: demographics and professional profile; access and technical competency; self-rated knowledge of indications, contraindications, and dosimetry; prescribing and perceived efficacy; attitudes toward phototherapy in the era of biologic and targeted therapies; and barriers and training needs. All 34 items were required; no incomplete responses were received. The complete questionnaire is provided in the Appendices.

Two guideline-based knowledge items with expected answers were included: (a) the maximum recommended cumulative number of PUVA sessions, for which 200 sessions represents a widely cited risk-monitoring threshold beyond which the probability of squamous cell carcinoma increases substantially, particularly in lighter phototypes (British Association of Dermatologists phototherapy guidelines; American Academy of Dermatology-National Psoriasis Foundation guideline [5,9]); and (b) the minimum recommended age for NB-UVB initiation, for which eight years is a commonly used practice threshold, while acknowledging that NB-UVB has been used in younger children under specialist supervision and that PUVA has different age-related considerations [5]. These two items provide a limited assessment of guideline-based knowledge and should not be interpreted as a comprehensive measure of operational or dosimetric proficiency.

Participant recruitment

The survey link was distributed via the WhatsApp messaging group (WhatsApp LLC, Menlo Park, CA, USA) of the Moroccan Society of Dermatology, which at the time comprised approximately 539 members (representing approximately 83% of the estimated 650 registered dermatologists in Morocco), and through the CHU Ibn Rochd dermatology network. Five reminders were sent over the six-week survey period. Participation was voluntary, anonymous, and unpaid. No formal sampling frame was applied; the survey therefore represents a convenience sample. Google Forms was set to limit submissions to one response per device; however, duplicate responses from different devices cannot be entirely excluded. Morocco has an estimated 650 registered dermatologists. Because the survey link may have been forwarded beyond the original distribution group, the exact denominator is uncertain; however, the minimum response rate was 53 of 539 (9.8%), and the maximum coverage of the profession was 53 of 650 (8.2%).

Statistical analysis

Categorical data are reported as counts and percentages. For multiple-response items, the denominator was the total sample (n = 53), so totals may exceed 100%. Exact 95% CIs (Clopper-Pearson method) were computed for the principal proportions. In addition, four pre-specified exploratory cross-tabulations were performed using Fisher’s exact test: access by practice setting (public/university vs private), phototherapy training during residency by self-reported ability to operate a unit, access by regular prescribing frequency, and any prior training by correct identification of the PUVA threshold. These analyses are exploratory; the study was not powered for inferential comparisons. Analyses were performed in Python 3.12 (pandas, scipy, and statsmodels).

## Results

Respondent characteristics

Fifty-three dermatologists responded. Most were female (90.6%). Respondents spanned all career stages: 17% were under 30 years, 35.8% were 30-40, 26.4% were 40-50, 3.8% were 50-60, and 17% were over 60. Private practice predominated (50.9%), followed by public (28.3%) and university (18.9%) settings. Weekly volume was moderate: 47.2% saw 50-100 patients, and 9.4% exceeded 100. The regional distribution showed a concentration in the Casablanca-Settat region (64.2%) and Rabat-Salé-Kénitra (18.9%), with smaller contributions from other regions (Table 1).

**Table 1:** Sociodemographic and professional characteristics of respondents (n = 53)

Variable	n	%
Age group		
<30 years	9	17
30-40 years	19	35.8
40-50 years	14	26.4
50-60 years	2	3.8
> 60 years	9	17
Sex		
Female	48	90.6
Male	5	9.4
Practice setting		
Private	27	50.9
Public	15	28.3
University	10	18.9
Mixed	1	1.9
Weekly patient volume		
<50/week	23	43.4
50-100/week	25	47.2
>100/week	5	9.4
Region		
Casablanca-Settat	34	64.2
Rabat-Salé-Kénitra	10	18.9
Marrakesh-Safi	2	3.8
Tangier-Tétouan-Al Hoceima	2	3.8
Souss-Massa	2	3.8
Guelmim-Oued Noun	1	1.9
Other/unspecified	2	3.8

Access and technical competency

Only 23 respondents (43.4%; 95% CI: 29.8-57.7%) had access to a phototherapy unit. NB-UVB was the most frequently mastered modality (66), then PUVA (62.3%) and excimer therapy (37.7%); three respondents (5.7%) reported no mastery of any technique. Asked whether they could independently run a phototherapy cabin, 58.5% said yes, 28.3% partially, and 13.2% no (Table 2).

**Table 2:** Access to phototherapy and technical competency (n = 53) NB-UVB, narrowband ultraviolet B; PUVA, psoralen plus ultraviolet A

Variable	n	%
Access to a phototherapy unit		
Yes	23	43.4
No	30	56.6
Modality mastered (multiple responses)		
NB-UVB	35	66
PUVA	33	62.3
Excimer	20	37.7
None of these	3	5.7
Able to independently run a phototherapy cabin		
Yes	31	58.5
Partially	15	28.3
No	7	13.2

Knowledge of indications, contraindications, and dosimetry

Most respondents rated their knowledge of indications as sufficient or better (77.4%) and of contraindications as sufficient or better (83%). Performance on the two guideline-based knowledge items was weaker. For the PUVA risk-monitoring threshold, only nine respondents (17%; 95% CI: 8.1-29.8%) identified 200 sessions; 20 (37.7%) answered “do not know,” and most others overestimated. For the minimum age for NB-UVB initiation, 18 (34%; 95% CI: 21.5-48.3%) identified eight years, whereas 19 (35.8%) chose 12 years (Table 3).

**Table 3:** Responses to the two guideline-based knowledge items (n = 53) NB-UVB, narrowband ultraviolet B; PUVA, psoralen plus ultraviolet A

Variable	n	%
Maximum cumulative PUVA sessions (guideline threshold: 200)		
50	1	1.9
100	7	13.2
150	5	9.4
200 (guideline threshold)	9	17
250	5	9.4
300	1	1.9
Do not know	20	37.7
Other/non-evaluable	5	9.4
Minimum recommended age for NB-UVB initiation (expected: eight years)		
4 years	4	7.5
Eight years (expected)	18	34
12 years	19	35.8
Do not know	8	15.1
Other/non-evaluable	4	7.5

Prescribing practices and perceived efficacy

Fifty respondents (94.3%; 95% CI: 84.3-98.8%) had prescribed phototherapy. Among prescribers, 38% prescribed regularly (> 20 patients/year), 26% occasionally (5-20), and 36% rarely (< 5). Perceived efficacy was high (79.2% effective, 13.2% very effective), and 86.8% (95% CI: 74.7-94.5%) perceived phototherapy as safe for their patients. The conditions most associated with phototherapy were psoriasis (96.2%) and vitiligo (92.5%), then chronic pruritus (84.9%), cutaneous lymphoma (75.5%), prurigo (69.8%), and atopic dermatitis (54.7%); morphea was recognized by 26.4% (Table 4).

**Table 4:** Prescribing practices, perceived efficacy, and associated indications (n = 53)

Variable	n	%
Ever prescribed phototherapy		
Yes	50	94.3
No	3	5.7
Prescribing frequency, among prescribers (n = 50)		
Regularly (>20/year)	19	38
Occasionally (5-20/year)	13	26
Rarely (<5/year)	18	36
Perceived efficacy		
Very effective	7	13.2
Effective	42	79.2
Little effective	4	7.5
Perceived safe for patients		
Yes	46	86.8
No	6	11.3
No response	1	1.9
Conditions associated with phototherapy (multiple responses)		
Psoriasis	51	96.2
Vitiligo	49	92.5
Chronic pruritus	45	84.9
Cutaneous lymphoma	40	75.5
Prurigo	37	69.8
Atopic dermatitis	29	54.7
Morphea	14	26.4

Phototherapy in the era of biologic and targeted therapies

Thirty-seven respondents (69.8%; 95% CI: 55.7-81.7%) held that phototherapy retains a major role despite the availability of biologic and targeted therapies, and none considered it obsolete. Most reported offering phototherapy before escalation: 83% (95% CI: 70.2-91.9%) before IL-17/IL-23 inhibitors in psoriasis, 75.5% (95% CI: 61.7-86.2%) before targeted therapy (including topical JAK inhibitors) in vitiligo, and 62.3% (95% CI: 47.9-75.2%) before dupilumab in atopic dermatitis (Table 5). It should be noted that in vitiligo, the comparator includes topical targeted therapies, which differ from the systemic biologic escalation relevant to psoriasis and atopic dermatitis. Several respondents identified the absence of a local phototherapy unit as the main driver of escalation to systemic therapy.

**Table 5:** Attitudes toward phototherapy in the era of biologic and targeted therapies (n = 53)

Variable	n	%
Role of phototherapy alongside biologic and targeted therapies		
A major role	37	69.8
A limited role	15	28.3
Obsolete	0	0
Unspecified	1	1.9
Phototherapy offered before escalation (yes)		
Psoriasis (before IL-17/IL-23 inhibitors)	44	83
Vitiligo (before targeted therapy including topical Janus kinase inhibitor)	40	75.5
Atopic dermatitis (before dupilumab)	33	62.3

Barriers and development needs

Logistical constraints (multiple sessions, travel, and scheduling) were the most common barrier (79.2%), followed by institutional barriers (45.3%) and absence of dedicated space (37.7%); low financial return and limited time were each cited by fewer than 10%. The leading development priorities were equipment (79.2%), training (58.5%), reimbursement reform (49.1%), and patient awareness (41.5%) (Table 6).

**Table 6:** Barriers to phototherapy practice and prioritized development needs (n = 53)

Variable	n	%
Barriers encountered (multiple responses)		
Logistical (sessions, travel, and scheduling)	42	79.2
Institutional (administrative and reimbursement)	24	45.3
Absence of dedicated space	20	37.7
Low financial return	3	5.7
Lack of time	2	3.8
Priority needs for development (multiple responses)		
Equipment acquisition	42	79.2
Structured training	31	58.5
Reimbursement reform	26	49.1
Patient awareness	22	41.5

Training background and continuing education needs

Only 35.8% had received dedicated phototherapy training during residency, and 49.1% had received none at all; 37.7% reported no structured training of any kind. Satisfaction with residency training was low, with 31 respondents (58.5%) reporting that they were not or only slightly satisfied (denominator: all 53 respondents, including four who did not answer). Knowledge sources were chiefly university training (77.4%), scientific meetings (43.4%), literature and internet (37.7%), and colleagues (20.8%). Interest in further training was near-universal (94.3%), with practical workshops preferred (64.2%), and 84.9% (95% CI: 72.4-93.3%) were willing to join a national or regional phototherapy network (Table 7).

**Table 7:** Training background and continuing education needs (n = 53)

Variable	n	%
Dedicated phototherapy training during residency		
Yes	19	35.8
Partial	8	15.1
None	26	49.1
Ever undertaken any structured phototherapy training		
Never	20	37.7
Satisfaction with residency training		
Very satisfied	4	7.5
Satisfied	14	26.4
Little satisfied	20	37.7
Not satisfied	11	20.8
No response	4	7.5
Main knowledge sources (multiple responses)		
University training	41	77.4
Scientific meetings	23	43.4
Literature and internet	20	37.7
Colleagues	11	20.8
Interested in further training		
Yes	50	94.3
No	3	5.7
Preferred training format (multiple responses)		
Practical workshops	34	64.2
Online seminars	22	41.5
Congress/continuing medical education	15	28.3
University diploma	13	24.5
Willing to join a national/regional network		
Yes	45	84.9
No	7	13.2
No response	1	1.9

Exploratory cross-tabulations

Access to a phototherapy unit was strongly associated with public or university practice settings compared with private practice (76% vs 14.3%; Fisher’s exact p < 0.001, OR = 19.0). Among those with access, 78.3% prescribed regularly, compared with only 3.3% of those without access (p < 0.001, OR = 104.4). Residency training was not significantly associated with self-reported ability to independently operate a phototherapy cabin (p = 0.39), and prior training of any kind was not significantly associated with correctly identifying the PUVA risk-monitoring threshold (p = 0.72). 

## Discussion

To our knowledge, this appears to be the first structured survey of phototherapy knowledge, perceptions, and practice among Moroccan dermatologists. We searched PubMed, Scopus, and Google Scholar (June 2026) using the terms “phototherapy” AND “Morocco” AND (“survey” OR “knowledge” OR “practice”) and identified no prior comparable studies. The findings have potential relevance both for local practice improvement and for the broader literature on phototherapy utilization in resource-limited settings with predominantly darker-skinned populations.

Respondents broadly valued phototherapy and reported offering it before biologic and targeted therapies across the three major indications surveyed. This pattern is consistent with current clinical guidelines: NB-UVB is recommended as first-line phototherapy for generalized vitiligo [10], and international guidelines, including those from NICE, position phototherapy as second-line therapy for moderate-to-severe psoriasis before biologic agents are considered [5,11]. The reported rates of phototherapy-first practice appear higher than in many high-income systems, where broader reimbursement of biologic and targeted therapies tends to shorten or bypass the phototherapy step. In the Moroccan context, cost considerations and a predominantly darker-skinned population in which phototherapy may be particularly relevant likely contribute to this stepwise approach.

The population-level relevance of phototherapy for Moroccan patients warrants discussion in the context of published literature while recognizing that our survey did not collect patient-level phototype, clinical outcome, adverse event, or cost data. In phototypes III-V, melanin acts as a UV filter, raising the doses needed for therapeutic efficacy and demanding careful dosimetric calibration [12]. Published observational data suggest that the PUVA-associated risk of non-melanoma skin cancer, a principal concern in long-term phototherapy in phototypes I-II, may be lower in more deeply pigmented skin, although this does not mean that cumulative carcinogenic risk is absent or uniformly reduced across all phototypes, modalities, and treatment regimens [4]. A recent systematic review confirmed high NB-UVB response rates for psoriasis in phototypes III-VI [13]. In vitiligo, NB-UVB, which mobilizes follicular melanocytes with favorable color matching, has largely replaced PUVA as first-line phototherapy [14]. These observations from the published literature provide a rationale for phototherapy in the Moroccan population, but further local studies collecting clinical outcome and safety data are needed.

The gaps in guideline-based knowledge identified in this survey merit attention, while acknowledging the limited scope of the two knowledge items. Only 17% of respondents identified 200 sessions as the PUVA risk-monitoring threshold, beyond which the risk of squamous cell carcinoma increases substantially in lighter phototypes [4,5,9], and more than one-third were unsure, suggesting insufficient coverage of PUVA dosimetry in training. However, it should be recognized that 200 sessions represent a risk-monitoring threshold rather than an absolute treatment maximum and that cumulative UVA dose, patient phototype, and individual risk factors are also relevant to treatment decisions. Regarding the minimum age item, 35.8% of respondents chose 12 years against an expected threshold of eight years for NB-UVB, but this threshold is not universally established across all guidelines, and NB-UVB has been used in younger children under specialist supervision. Overestimation of the minimum age may nevertheless contribute to delayed initiation of phototherapy in pediatric vitiligo, where earlier intervention is generally associated with better repigmentation outcomes.

The training landscape is structurally deficient: nearly half received no dedicated phototherapy training during residency, and most were dissatisfied with the training they did receive. The same deficit has been documented in high-income settings, where both residency program directors and trainees judge phototherapy to be inadequately taught [15,16]. This contrasts with formal accreditation standards for phototherapy units in the United Kingdom [9] and competency frameworks from the European Society for Photodermatology. Without comparable standards and with a private sector expanding faster than public phototherapy infrastructure, phototherapy expertise risks progressive dilution across successive cohorts. This is a self-reinforcing cycle, in which less training begets less practice and further erosion of expertise, and it has already produced a measurable decline in phototherapy use even in well-resourced health systems [17]. In a resource-limited setting, this trajectory has particular implications, as the relative affordability of phototherapy makes its preservation relevant to equitable access to dermatological care.

The barriers identified in this survey mirror those in other LMICs. Logistical constraints such as repeated attendance, travel, and scheduling conflicts disproportionately affect patients with limited transport and without employer-covered leave. Home-based phototherapy, shown to be non-inferior to office treatment in plaque psoriasis [18], and decentralized regional units could help mitigate these barriers. The exploratory finding that access to a phototherapy unit was the strongest determinant of regular prescribing (OR = 104.4, p < 0.001) reinforces the view that equipment availability, rather than attitude, is the primary constraint on phototherapy use.

That 84.9% of respondents would join a national phototherapy network is noteworthy. Such a network could facilitate standardization of dosimetric protocols, multicenter audits, continuing education, and the collection of outcome data needed to inform equipment deployment and reimbursement policy.

This study has several limitations that should be considered when interpreting the findings. The sample of 53 respondents was obtained through convenience sampling via professional networks; the distribution group comprised approximately 539 of the estimated 650 registered dermatologists in Morocco (83% coverage), yielding a minimum response rate of 9.8% (53/539). Respondents were geographically concentrated in the Casablanca-Settat (64.2%) and Rabat-Salé-Kénitra (18.9%) regions, which may limit the generalizability of findings to rural or underserved areas and introduce potential selection, non-response, and volunteer bias. Although Google Forms was configured to limit submissions to one per device, duplicate responses from different devices cannot be entirely excluded. Knowledge was assessed using only two guideline-based items, which are insufficient to capture overall operational or dosimetric proficiency; the responses to these items may depend on the specific modality, guideline, patient age, and clinical context. The questionnaire underwent face validation only and was not formally psychometrically validated. All competency, efficacy, and safety assessments were self-reported and may not reflect objectively measured clinical performance or actual patient outcomes. No patient-level data on phototype distribution, clinical outcomes, adverse events, cumulative UV dose, or cost-effectiveness were collected. The cross-sectional design precludes causal inference. The study should not be interpreted as a nationally representative assessment of Moroccan dermatological practice.

## Conclusions

Moroccan dermatologists express strong support for phototherapy and report offering it before biologic and targeted therapies across the major indications surveyed, but deficits in equipment access, guideline-based knowledge, and postgraduate training represent important barriers to optimal practice. Access to a phototherapy unit was the strongest determinant of regular prescribing, and existing training did not appear to ensure familiarity with key dosimetric thresholds. These findings highlight the need for further research, including studies that objectively assess clinical competency and collect patient-level outcome and safety data. Interventions that merit investigation include phased equipment deployment to underserved public hospitals, integration of structured phototherapy training into residency curricula, and the establishment of a national phototherapy network for continuing education, protocol standardization, and epidemiological surveillance.

## Appendices

https://docs.google.com/forms/d/e/1FAIpQLSf9dBheoCWS32rxWZ1QcaXCmgSihJ_0im0-R-C6riuaBtDmWA/viewform
